# Periodic Breathing and Behavioral Awakenings at High Altitude

**DOI:** 10.1155/2015/279263

**Published:** 2015-09-21

**Authors:** Daniel J. Shogilev, John B. Tanner, Yuchiao Chang, N. Stuart Harris

**Affiliations:** ^1^Division of Emergency Medicine, Duke University, Durham, NC, USA; ^2^Division of Wilderness Medicine, Department of Emergency Medicine, Massachusetts General Hospital, Boston, MA, USA; ^3^Division of General Internal Medicine, Massachusetts General Hospital, Boston, MA, USA

## Abstract

*Objectives*. To study the relationship between nocturnal periodic breathing episodes and behavioral awakenings at high altitude. *Methods*. Observational study. It is 6-day ascent of 4 healthy subjects from Besisahar (760 meters) to Manang (3540 meters) in Nepal in March 2012. A recording pulse oximeter was worn by each subject to measure their oxygen saturation and the presence of periodic breathing continuously through the night. An actigraph was simultaneously worn in order to determine nocturnal behavioral awakenings. There were no interventions. *Results*. 187-hour sleep at high altitude was analyzed, and of this, 145 hours (78%) had at least one PB event. At high altitude, 10.5% (95% CI 6.5–14.6%) of total sleep time was spent in PB while 15 out of 50 awakenings (30%, 95% CI: 18–45%) occurring at high altitudes were associated with PB (*P* < 0.001). *Conclusions*. Our data reveals a higher than expected number of behavioral awakenings associated with PB compared to what would be expected by chance. This suggests that PB likely plays a role in behavioral awakenings at high altitude.

## 1. Introduction

Sleep disturbances in response to acute altitude ascent are very common and well documented. Objective changes in sleep architecture were first documented by Joern et al. in 1970 in a study of two men at greater than 3000 m. in Antarctica. The study revealed a near absence of deep wave sleep, a substantial decrease in rapid eye movement (REM) sleep, and the presence of periodic breathing (PB) with associated arousals [1]. Further studies have also shown that sleep at high altitude is characterized by an increase in arousals and awakenings, nocturnal hypoxemia, and a general diminished quality [2].

This study seeks to further contribute to the understanding of high-altitude sleep by focusing on PB. PB, when occurring in healthy individuals, almost universally predominates at high altitudes [2–4]. It is characterized by a sporadic waxing and waning breathing pattern with shifting periods of hyperventilation and central hypopnea and even apnea. Much controversy surrounds the role of PB at high altitudes and sleep disturbances. While some studies and reviews suggest an intimate correlation of periodic breathing and arousals during sleep at high altitudes [3, 5], others have failed to find a significant association between periodic breathing and high-altitude sleep disturbances [6–8].

In this present study, we sought to test the hypothesis that the PB events at elevated altitude are associated with an increase in behavioral awakenings. In doing so, we hope to clarify how PB potentially influences high-altitude sleep.

## 2. Methods

### 2.1. Participants, Study Design, Setting, and Ascent Profile

**Figure 1:**
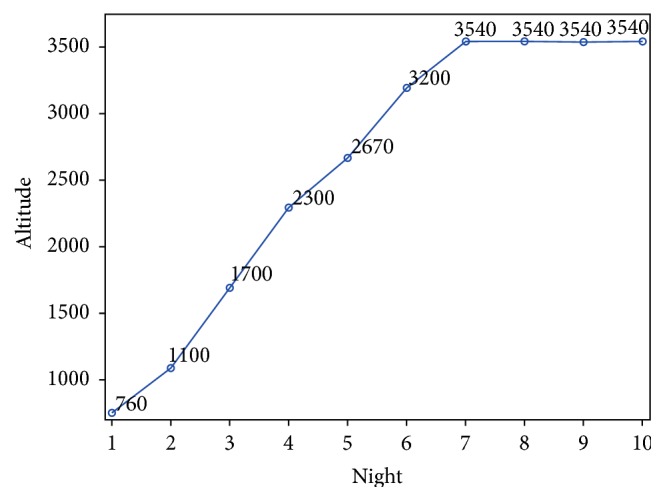
Ascent profile.

To test this hypothesis we performed nocturnal measurements of PB and wakefulness of four trekkers during a 6-day ascent from 760 meters to 3540 meters. This prospective study was carried out by medically savvy volunteers in March 2012.  Table 1 shows the demographic profile of the four trekkers. The starting point of the trek was at Besisahar at an altitude of 760 meters. The peak altitude was reached on day 6 at Manang (3540 meters). The trekkers were monitored for 4 additional nights at this peak altitude.  Figure 1 shows the complete ascent profile.

No interventions, invasive measures, or medications were used during this trek. As such, Institutional Review Board (IRB) approval was not obtained.

### 2.2. Study Measurements and Statistical Methods

#### 2.2.1. Actigraphy

An actigraph differentiates sleep from wakefulness based on wrist movement. Estimation of sleep by actigraphy is highly correlated with sleep estimation by polysomnography [9–11] and is a validated utility for sleep research at high altitude [12, 13]. The particular actigraph used for this study was the Motion Logger (Ambulatory Monitoring Inc., Ardsley, NY, USA). Study subjects wore the actigraph on the wrist of their dominant hand. Subjects pressed an event button when they laid down to sleep and again when they awoke in the morning. Recordings between the two events were analyzed to detect awakenings (as determined by the Cole-Kripke algorithm) [9]. In addition, a pulse oximeter was used to measure the SaO_2_ of the trekkers, the details of which are discussed below. This methodology enabled us to discern the exact time at which awakenings and PB events occurred. On the first day of the study, the actigraph and pulse oximeter were set to the same exact time in an effort to identify any temporal correlation between awakenings as determined by actigraphy and PB events as determined by pulse oximetry. Data were recorded using zero crossing mode (ZCM) in one-minute epochs. Automated analysis was performed using the Cole-Kripke algorithm on the ZCM channel at a one-minute sample rate (Action 4 Version 1.13, Ambulatory Monitoring Inc., Ardsley, New York) [9, 10, 14].

#### 2.2.2. Pulse Oximetry

A recording pulse oximeter worn on the nondominant hand was used to measure SaO_2_ and heart rate (Nonin 3100 WristOx, Nonin Medical Inc., Plymouth, MN, USA). Study subjects were instructed to put on the pulse oximeter when they went to bed and to remove the pulse oximeter the next morning when they arose. Time in periodic breathing was determined manually from periodic desaturations in a crescendo-decrescendo pattern lasting at least 3 cycles [12] and having an amplitude of 4% or greater. An awakening was considered to be associated with PB if a PB event occurred within one minute prior to the awakening. In our data analysis, the raw pulse oximetry data were first amended by excluding segments of less than 5 minutes of continuous pulse oximetry recordings. We believe that short, interrupted segments of pulse oximetry data are too brief to allow reliable interpretation. To maximize data quality, we adopted a conservative approach, only analyzing segments of 5 minutes or greater. All remaining tracings were analyzed to compute total minutes spent in periodic breathing.  Table 2 shows the number of hours each subject contributed to this sleep study.

Overall, 358-hour raw data were available for analysis. The raw hourly data were further amended to 285 hours of analyzable data using the following hierarchy of exclusions: (1) nights with less than 200 minutes of pulse oximetry recording as this fragment was not likely to represent an adequate sleep sample and thus may omit the potential variations in breathing that occur during the different sleep stages throughout the night, (2) hours excluded after pulse oximetry measurements ended but actigraph was still on, and (3) final hour of sleep or the final hour of night. This final criterion was adopted because the final hour of sleep naturally includes a terminal awakening which may have distinct physiological and adaptive causes in comparison to midsleep awakenings [15]. This allowed us to better focus on the potential correlation between awakenings and periodic breathing.

#### 2.2.3. Statistical Analysis

For the purpose of this study, we defined “low altitude” to be <2670 meters and “high altitude” to be ≥2670 meters. This altitude was reached on the 6th day of the trek. This was chosen as the cut-off level for multiple reasons. Most notably, consensus high altitude scoring systems recognize elevations above 2,500 meters as those where the pernicious physiologic effects of acute high altitude become increasingly common and severe [16].  Table 3 lists the mean oxygen saturations as well as time spent in PB at each given altitude during ascent. This was borne out in our own data, as >2670 m was the initial altitude at which a substantial decrease in SaO_2_ was observed. Additionally, subsequent nights at higher altitudes had substantial increases in the amount of both PB events and desaturations. For these reasons, only data obtained at 2670 m and higher was used in our statistical analysis of “high altitude” events.

Data were collected from each of the 4 subjects for a total of 10 nights (4 nights at low altitude and 6 nights at high altitude). During each night, evaluation on PB events and awakenings was done on the hourly basis. Depending on the nature of the parameter, data were analyzed by night (e.g., percentage of the night spent in PB) or by hour (e.g., PB or awakening event). Repeated measures analyses using Generalized Estimating Equations (GEE) techniques were used to take into account the multiple hourly or nightly measurements from the same subject. Statistical analysis was performed using SAS version 9.3 (The SAS Institute, Cary, NC).

## 3. Results

### 3.1. Duration of PB Events (Nightly Data)

Among the six nights spent at high altitude, the percentage of the night spent in PB ranged from 0.7% (subject 2 on night 5) to 34.4% (subject 1 on night 10). When averaging across the six nights at high altitude, the four subjects spent 21.8%, 4.1%, 15.2%, and 2.9% of the night in PB, respectively. When averaging across all nights at high altitude, subjects spent an average of 10.5% ± 9.4% of total sleep time in PB compared to 2.4% ± 2.4% while at low altitudes.

### 3.2. Periodic Breathing Associated Awakenings (Hourly Data)

An awakening was categorized as a PB-associated awakening if there was a PB event at any point during the one minute prior to the awakening. Actigraphy calculated a total of 50 behavioral awakenings for all four climbers over the course of 6 nights at high altitude. 15 out of 50 of these awakenings (30%, 95% CI: 18–45%) were associated with PB (Table 4). As indicated above, subjects spent an average of 10.5% of total sleep time in PB while at high altitude. Therefore, the occurrence of a PB-associated awakening event was significantly higher than what would be expected by chance (30% versus 10.5%, *P* < 0.001).

## 4. Discussion

Decreased sleep quality is a near-universal finding at high altitude. While a significant component of the disruption in sleep architecture at high altitude is due to an increased incidence of PB, the role of PB's influence on behavioral awakenings is poorly defined. This study presents data to further our understanding of this relationship.

In this study, we use actigraphy to capture data on patients' behavioral awakenings. In contrast, many prior studies use polysomnography instead. Polysomnography is the gold standard for sleep studies [17] against which actigraphy is measured. It is a labor intensive measure of sleep states and architecture that uses both electrophysiological and behavioral components, whereas actigraphy uses a less invasive method to measure sleep/wake cycles based on gross motor movements. The argument in favor of actigraphy is that the motor activity it measures is a common feature to the sleep-wake state, and thus its presence or absence can be used to accurately estimate these parameters [17]. This assertion is not universally endorsed [18]. Several studies have shown that actigraphy is very sensitive but nonspecific for detecting sleep and wakefulness [11, 17, 19–21]. In short, the ability of actigraphy to detect wakefulness is relatively poor, but the overall accuracy of actigraphy in detecting sleep and wakefulness is relatively high [11, 17, 19, 20]. Since accuracy is determined by both sensitivity and specificity, these test characteristics make actigraphy tend to overestimate sleep and underestimate wakefulness [19, 21]. This is likely because actigraphy determines behavioral awakenings based on gross movement and may fail to recognize awakenings or arousals that occur with minimal or no movement [17].

In our study, we recognize the aforementioned limitations of actigraphy. For this reason, our study was not powered to evaluate sleep architecture, sleep quality or to determine the absolute number of awakenings that occurred during sleep. Rather, we simply used actigraphy as a tool to determine and analyze only a proportion of the total awakenings (behavioral) that occurred at night.

Furthermore, the majority of polysomnography data on sleep at high altitudes measures arousals and the Arousal Index (AI), the amount of arousals per hour. We, however, measured discrete behavioral awakenings as determined by actigraphy. Although these two terms are often used interchangeably in the literature, this is technically imprecise. One definition asserts that an “arousal” indicates cortical and physiological events linked to respiratory pathology whereas an “awakening” also indicates a behavioral element [15]. Arousals represent transitions from deeper to lighter sleep with the progression to a full awakening as a potential outcome [15]. Since there is an unquantifiable relationship between the aforementioned concepts, it is impossible to compare the two, and we acknowledge that our findings on awakenings are not generalizable to those that specifically measure arousals.

We examined the potential relationship between PB and behavioral awakenings by reviewing the minute prior to each awakening for evidence of a PB event. At high altitude, the average percent of time spent in PB among the 4 subjects was 10.5% (95% CI 6.5–14.6%). Interestingly, out of the 50 measured awakenings at high altitude, 30% were associated with periodic breathing (95% CI: 18–45%; *P* = 0.02). Thus, the number of associated awakenings is not explained by chance alone, as it is significantly higher than 10% expected (based on percent of the night spent in periodic breathing). As such, it is likely that PB may play at least some role in awakenings at high altitude. We followed the recommended protocol and used one-minute actigraphy intervals (for purposes of conserving battery and memory). Some literature indicates that the one-minute epochs available from actigraphy may be too long to accurately determine a potential correlation between PB and behavioral awakenings. Weil and Salvaggio et al. argued that the mechanism of PB that potentially leads to arousals or awakenings is closely related to the termination of apnea and the initiation of hyperpnoea [5, 22]. Pack et al. analyzed arousals occurring within 5 seconds of the termination of the PB event it was attributed to [23] and Khoo et al. only used 1-second intervals and argued that the 5-second interval used by Pack et al. and earlier studies was inadequate to determine the relationship of PB with arousals [24]. Our algorithm included any periodic breathing in the minute prior to the awakening and would pick up PB that was occurring in the seconds immediately prior to the awakening. As such, we believe our findings have merit.

As previously stated, the majority of research on this topic looks at the relationship between periodic breathing and arousals and not awakenings. Johnson et al. and Salvaggio et al. both showed that while the total number of arousals tends to increase with the incidence of PB events, a large proportion of PB events do not elicit arousals [3, 22]. In Johnson et al.'s study, subjects aroused to only half of the apnea and hypopnea cases [3]. Conversely, in a 2012 study by Nussbaumer-Ochsner et al. of 16 healthy mountaineers who spent one night at an altitude of 3610 meters and then four subsequent nights at an altitude of 4559 meters, they found that on the first night at an altitude of 4559 meters only 11% of apnea/hypopnea cases were followed by an arousal and on the third night at this altitude this decreased to 4%. During this time, the apnea-hypopnea index (AHI) (a measure of the frequency of PB episodes) increased while the AI decreased, undermining the potential for a causal relationship between PB and arousals [7]. They found that sleep disturbances were more closely related to hypoxemia rather than PB [7].

These mixed conclusions illustrate the inherent difficulty in understanding the interrelationships between PB, arousals, awakenings, and other sleep disturbances. Some have suggested that PB lowers the threshold to arousals or awakenings. Among others, Khoo et al. have reported [24] that increased arousals destabilize breathing and lead to PB. Alternatively, hypoxia may be the common cause of both PB and arousals with PB and arousals and awakenings being otherwise unrelated.

Weil postulated that PB during acclimatization at high altitudes likely reflects an individual's ventilatory response to hypoxia. As such, there is great variability in the individual response to altitude [5]. Our study reflects this intersubject variability as our four subjects spent an average of 21.8%, 4.1%, 15.2%, and 2.9% in PB at high altitudes, respectively.

## 5. Limitations

There are several other limitations to the findings of this study. As with many studies on sleep at high altitude, we had a very small sample size. This limited the power of our study and made our results vulnerable to the effect of potential outliers. In the present study, two of the subjects contributed 83% of the time spent in periodic breathing. In order to adequately analyze the data obtained from a small group of subjects and compensate for individual variation, we studied each hour of sleep and each awakening as an isolated event. With a large number of hours and discrete events to study we were able to describe statistically significant findings which contribute to the growing body of research on this matter. Nonetheless, further study should be performed with larger sample sizes. Since this study was conducted at a moderately high altitude and gradual ascent rate, our findings cannot be generalized to scenarios or varying ascent rates and commonly mountaineered higher altitudes such as Mt. Kilimanjaro, the Andes, and Mt. Everest. Lastly, pharmacologic sleep aids such as Temazepam or Acetazolamide are commonly used during high altitude ascent. No such medications were used by the participants in this study, and, thus, it does not shed light on the relationship between PB and awakenings when these medications are used.

In the future, we believe it would be beneficial to further examine the potential mechanisms behind why PB may lead to a more wakeful state. Additionally, since much of the data are conflicting and all studies take place in unique settings, including different ascent rates and peak altitudes, varying weather conditions, and the use of pharmacologic sleep aids, we believe that a more controlled study (where a greater sample size would likely be more readily available) may help obviate any apparent inconsistences in the observed results and conclusions reached.

## 6. Conclusion

In conclusion, our data reveal a higher than expected number of awakenings that are associated with a PB. This finding suggests that PB plays a role in awakenings at high altitude.

## Figures and Tables

**Table 1:** Trekker demographics.

Subject	Gender	Age	Height (m)	Weight (kg)	BMI (kg/m^2^)	Sleep past medical history	Medications on trek
1	M	56	1.75	74	24.2	No	No
2	F	40	1.6	56	21.9	No	No
3	M	31	1.9	82	22.7	No	No
4	F	26	1.65	57	20.9	No	No

**Table 2:** Contribution of subjects to sleep study.

Subject	Total hours of raw sleep data	Total hours of sleep analyzed	Total hours of sleep excluded	Hours excluded owing to less than 200 minutes of pulse oximetry recording	Hours excluded after pulse ox ended but actigraph still on/last hour of the night	Total number of awakenings	Total hours of periodic breathing
1 (E)	88	60	28	9	19	21	10.8
2 (K)	91	73	18	8	10	11	2.4
3 (J)	91	80	11	0	11	29	8.4
4 (S)	88	72	16	6	10	14	1.7
TOTAL	**358**	**285**	**73**	**23**	**49**	**75**	**23.2**

**Table 3:** Average O_2_ saturation and percent of night spent in periodic breathing.

Altitude (m)	Avg O_2_ saturation (%)	% night in PB
760	95.6	1.2
1100	95.5	3.1
1700	95.4	1.7
2300	93.3	3.5
2670	91.0	5.3
3200	88.4	10.1
3540	87.2	13.0

**Table 4:** Relationships between awakenings and associated periodic breathing events.

Altitude (meters)	Awakening not associated with PB (number/%)	Awakening associated with periodic breathing (number/%)	Total	% night spent in PB
<2670 m	21 (91.3)	2 (8.7)	23	2.4
≥2670 m	35 (70.0)	15 (30)	50	10.5
Total	56	17	73	7.6
